# Reduced Retinal Microvascular Perfusion in Patients With Stroke Detected by Optical Coherence Tomography Angiography

**DOI:** 10.3389/fnagi.2021.628336

**Published:** 2021-04-13

**Authors:** Baoyi Liu, Yijun Hu, Guixian Ma, Yu Xiao, Bin Zhang, Yingying Liang, Pingting Zhong, Xiaomin Zeng, Zhanjie Lin, Huiqian Kong, Guanrong Wu, Zijing Du, Ying Fang, Manqing Huang, Lijuan Wang, Xiaohong Yang, Honghua Yu

**Affiliations:** ^1^Department of Ophthalmology, Guangdong Eye Institute, Guangdong Provincial People's Hospital, Guangdong Academy of Medical Sciences, Guangzhou, China; ^2^The Second School of Clinical Medicine, Southern Medical University, Guangzhou, China; ^3^Refractive Surgery Center, Aier Institute of Refractive Surgery, Guangzhou Aier Eye Hospital, Guangzhou, China; ^4^Aier School of Ophthalmology, Central South University, Changsha, China; ^5^Department of Neurology, Guangdong Neuroscience Institute, Guangdong Provincial People's Hospital, Guangdong Academy of Medical Sciences, Guangzhou, China

**Keywords:** optical coherence tomography angiography, retianal vascular, retinal vessel analysis, retina, stroke

## Abstract

Currently there is a shortage of biomarkers for stroke, one of the leading causes of death and disability in aging populations. Retinal vessels offer a unique and accessible “window” to study the microvasculature *in vivo*. However, the relationship between the retinal microvasculature and stroke is not entirely clear. To investigate the retinal microvascular characteristics in stroke, we recruited patients with stroke and age-matched control subjects from a tertiary hospital in China. The macular vessel density (VD) in the superficial capillary plexus (SCP) and deep capillary plexus (DCP), foveal avascular zone (FAZ) metrics, and optical coherence tomography angiography (OCTA) measured optic disc VD were recorded for analysis. A total of 189 patients with stroke and 195 control subjects were included. After adjusting for sex, visual acuity, systolic and diastolic blood pressure, a history of smoking, levels of hemoglobulin (HbA1c), cholesterol, and high-density lipoprotein (HDL), the macular VD of SCP and DCP in all sectors was decreased in patients with stroke. In the stroke group, the VD around the FAZ and the VD of the optic disk were lower. Logistic regression found the parafovea-superior-hemi VD of DCP > 54.53% [odds ratio (OR): 0.169] as a protective factor of stroke. Using the integration of all OCTA parameters and traditional risk factors, the area under the receiver operating characteristic (AUC) curve of distinguishing patients with stroke was 0.962, with a sensitivity of 0.944 and a specificity of 0.871. Our study demonstrates that the retinal VD is decreased in patients with stroke independently of the traditional risk factors of stroke, which may shed light on the monitoring of stroke using the retinal microvascular parameters.

## Introduction

Stroke is one of the leading causes of death and disability in aging populations, and places a remarkable burden on families and society (Feigin et al., 2016; Sarfo et al., 2018; Gregori-Pla et al., 2019). Every year ~800,000 people experience a stroke attack, with an estimated cost to the country of $34 billion in the USA (Benjamin et al., 2017). There is still a shortage of vascular biomarkers for stroke despite major advancements in neuroimaging technologies in recent years (Dieleman et al., 2014; Goubran et al., 2019; Karbasforoushan et al., 2019). Digital subtraction angiography (DSA) is the golden standard to evaluate the cerebral vasculature in patients with stroke (GA, 1992). However, invasive and radiation-exposed DSA examinations possibly result in allergies to the contrast agents (Bash et al., 2005). Retina offers a unique and accessible “window” to study the microvasculature *in vivo*. Because the retinal vasculature shares a similar embryological origin, anatomical features, and physiological properties with the brain vasculature (London et al., 2013; Jung et al., 2019; McGrory et al., 2019), retinal vessels may reflect the changes in cerebral vessels. Previous studies have shown the associations between stroke and changes of the retinal vasculature, including arterial narrowing, venous dilating, arteriovenous nipping, and vessel tortuosity, using a computer software analysis on color fundus images (Ong et al., 2013; Cheung et al., 2017; McGrory et al., 2019). However, a fundus photograph-based image analysis is rough, imprecise, and time-consuming.

Optical coherence tomography angiography (OCTA) is an automated and non-invasive examination that can demonstrate retinal vessel densities (VD) and the structure of foveal avascular zone (FAZ). In recent years, OCTA has been widely used to observe the retinal vasculature (Kashani et al., 2017; Ang et al., 2018). With the provision of ultrahigh resolution details of the retinal microvasculature, as well as the quantitative data of different vascular layers of retina, OCTA may be also useful in the evaluation of the retinal vasculature in patients with stroke (Huang et al., 2016).

In this prospective cross-sectional study, we used OCTA to quantitatively measure the retinal microvasculature, aiming to detect the changes in the retinal microvascular perfusion in patients with stroke.

## Materials and Methods

### Design and Participants

A prospective cross-sectional study was conducted in the Department of Neurology of Guangdong Provincial People's Hospital from May 2019 to December 2019. Patients who were hospitalized for the assessment of suspected stroke were scheduled for an examination using CT or MRI. Patients were consecutively included after the diagnosis of stroke was confirmed. The included criteria were: (1) confirmation of the diagnosis of stroke using CT or MRI (Klijn and Hankey, 2003); (2) a reliable ophthalmic examination with interpretable retinal OCTA data; (3) a normal intraocular pressure (IOP) (10–21 mmHg); and (4) an agreement to participate in the study.

The exclusion criteria were: (1) unstable vital signs; (2) a history of retinal diseases (such as hypertensive retinopathy or diabetic retinopathy (DR), vascular or degenerative macular diseases), uveitis, or glaucoma; (3) the presence of corneal or lens opacity compromising the image quality of the OCTA examination; (4) a poor eye fixation or poor cooperation to the ophthalmic examinations; and (5) a moderate or high refractive error (≥ + 3 diopters or ≤ −3 diopters) (Zeller et al., 2005).

In addition, 195 age-matched non-stroke volunteers (without either a history of stroke nor eye disease) were also included. The study was approved by the Research Ethics Committee of Guangdong Provincial People's Hospital [registration number: GDREC2018148H(R1)] and was performed according to the Declaration of Helsinki. Informed consent was obtained from all participants before the examination.

### Ophthalmic Examinations

All participants underwent the following ophthalmic examinations: an uncorrected visual acuity, the best-corrected visual acuity (BCVA) in decimals, IOP, a slit-lamp examination, fundus biomicroscopy, and the OCTA examination (RTVue-XR Avanti; Optovue, Fremont, CA, USA, version 2016.2.035). All OCTA examinations were performed by trained ophthalmologists in a dark room. The OCTA instrument is a spectral-domain OCT (Agemy et al., 2015; Spaide et al., 2015a,b) device, and retinal vascular images were generated using a split-spectrum amplitude decorrelation angiography algorithm (Motaghiannezam and Fraser, 2012). Two sets of 6 × 6 mm ultrahigh resolution scanning were performed for each eye at one section. Each scanning set comprised two raster volumetric patterns (one vertical and one horizontal). An orthogonal registration algorithm (built-in software to correct the motion artifacts) was used to merge the three-dimensional OCT angiograms. The VD (%) was defined as the percentage of signal positive pixels per total pixels in an area of interest. Superficial and deep retinal vascular networks were generated by using an automated software algorithm. The boundaries for each layer were as follows: a slab extending from 3 to 15 μm from the internal limiting membrane was generated for detecting the superficial capillary plexus (SCP) and a slab extending from 15 to 70 μm below the internal limiting membrane for the deep capillary plexus (DCP). The area (mm^2^), perimeter (mm), and acircularity index (the ratio between the measured perimeter and the perimeter of the same size circular area) of FAZ, the VD within a 300-mm ring surrounding FAZ (FD-300, %), and the optic disc VD of the whole image and capillary were obtained automatically by using the AngioVue software. Both eyes of the participants were examined using OCTA but only the data of the right eye were included. If the scan of the right eye was uninterpretable, data of the left eye were used. Only images with quality index ≥ 6 were retained.

### Data Extraction and Processing

Demographic and blood test data, CT/MRI reports, and ophthalmic data were extracted and recorded from the hospital registration system (version 3.0) by trained research assistants and ophthalmologists. The demographic and blood test data included patients' sex, age, duration of stroke, previous history (hypertension, diabetes mellitus, hyperlipidemia, coronary artery disease, atrial fibrillation, and the use of corresponding therapeutic drugs, including antihypertensive drugs, hypoglycemic agents, statin medications, aspirin and anticoagulant therapy), height, weight, blood pressure, laboratory test data [fasting blood glucose (FBG), glycated hemoglobulin (HbA1c), total cholesterol (CHOL), triglyceride (TRIG), non-esterified fatty acid (NEFA), high-density lipoprotein (HDL), low-density lipoprotein (LDL), d-dimer (D-DI), and homocysteine (HCY)]. Ophthalmic data included patients' ocular disease history, VA, BCVA, IOP, and the digital data extracted from the OCTA instrument. Key variables (CT/MRI details and OCTA parameters) were double recorded by two Chinese board-certified ophthalmologists (BL and YX), and inconsistent data were arbitrated by a retinal specialist (HY) to generate a final record.

### Statistical Analysis

Statistical analysis was performed by using the SPSS software package version 20 (SPSS. Inc., Chicago, IL, USA). Qualitative variables were presented as numbers and percentage. Quantitative variables were presented as mean (SD). Data normality were assessed by using the Shapiro–Wilk test. Two-tailed independent Student's *t*-test was applied to compare the basic characteristics between the stroke and control subjects. By comparisons of OCTA parameters, significantly different baseline characteristics between the two groups were adjusted. Baseline and OCTA parameters were also analyzed by using the univariate regression with stroke/control as a dependent variable. The OCTA parameters, which were statistically significant in the univariate regression, were divided into four categories: SCP, DCP, FAZ, and optic disc. The OCTA parameters with the highest odds ratios (ORs) in each category were entered into the multivariate binary regression model, together with the statistically significant baseline characteristics in the univariate regression, to explore the independent risk/protective factors of stroke. The outcomes of these regression analyses were expressed as ORs, CI stated at 95%, and *p*-value. Finally, a receiver operating characteristic (ROC) analysis was carried out to determine the performance of OCTA parameters to distinguish patients with stroke. The area under the ROC curve (AUC) was calculated. The optimal cutoff value was determined by the highest Youden index (sensitivity+specificity-1), and the corresponding sensitivity and specificity were recorded. Values of *p* were expressed as outcomes and a *p* < 0.05 was considered as statistically significant.

## Results

### Study Population

Overall, 189 patients with stroke (176 patients with ischemic stroke, 9 patients with hemorrhagic stroke, and 4 patients with indeterminate stroke) and 195 age-matched control subjects were included. The mean ± SD of age in the stroke group and the control group was 61.95 ± 11.99 years and 61.67 ± 8.40 years, respectively (*p* = 0.795). Demographic and clinical data of the two groups are shown in Table 1. The time gap between the first stroke attack and the OCTA examination was 1–419 days in the stroke group with the median of 13 days, and the time gap between the last stroke attack and the OCTA examination was 1–183 days in the stroke group with the median of 11 days. There were significant differences between the two groups in sex (*p* < 0.001), systolic blood pressure (SBP) (*p* < 0.001), diastolic blood pressure (DBP) (*p* < 0.001), a history of smoking (*p* < 0.001), HbA1c (*p* = 0.038), CHOL (*p* < 0.001), HDL (*p* < 0.001), and BCVA (*p* < 0.001).

**Table 1:** Demographic and clinical data of the two groups (*n* = 384).

**Characteristic, mean (SD)^a^**	**Stroke**	**Control**	***p*-value**
Number of eyes (patients)	189	195	N/A
**NEUROLOGICAL CHARACTERISTICS**			
Age, y^a^	61.95 (11.99)	61.67 (8.40)	0.795
Sex, female, n (%)	47 (24.87)	127 (65.13)	<0.001^*^
SBP at admission, mmHg	145.70 (21.69)	133.35 (16.07)	<0.001^*^
DBP at admission, mmHg	84.35 (11.74)	78.31 (10.89)	<0.001^*^
Duration of stroke (from the first attack), day	13 (1–419)	N/A	N/A
Duration of stroke (from the last attack), day	11 (1–183)	N/A	N/A
**PREVIOUS HISTORY**			
History of HTN, n (%)	88 (46.56)	90 (46.15)	1.000
History of DM, n (%)	103 (54.50)	88 (45.13)	0.101
History of HLP, n (%)	91 (48.15)	77 (39.49)	0.088
History of CAD, n (%)	9 (4.76)	4 (2.05)	0.167
History of AF, n (%)	2 (1.06)	N/A	N/A
History of smoking, n (%)	58 (30.69)	29 (14.87)	0.003^*^
**HISTORY OF THERAPIES**			
Antihypertensive drugs, n (%)	61 (32.28)	80 (41.03)	0.076
Hypoglycemic agents, n (%)	53 (28.04)	72 (36.92)	0.064
Statin medications, n (%)	7 (3.70)	14 (7.18)	0.135
Aspirin, n (%)	12 (6.35)	15 (7.69)	0.608
Anticoagulant therapy, n (%)	2 (1.06)	N/A	N/A
**LABORATORY TESTS**			
FBG, mmol/L	5.84 (2.53)	5.89 (2.43)	0.890
HbA1c, %	6.48 (1.63)	6.15 (1.05)	0.038^*^
CHOL, mmol/L	4.34 (1.15)	4.82 (1.12)	<0.001^*^
TRIG, mmol/L	1.64 (0.75)	1.59 (1.13)	0.630
NEFA, mmol/L	0.45 (0.19)	0.48 (0.19)	0.490
HDL, mmol/L	1.01 (0.23)	1.22 (0.25)	<0.001^*^
LDL, mmol/L	2.84 (0.89)	2.73 (1.27)	0.359
D-DI, ng/mL	636.64 (920.75)	482.27 (321.07)	0.437
HCY, μmol/L^b^	12.81 (5.40)	N/A^b^	N/A
**OPHTHALMOLOGICAL CHARACTERISTICS**			
BCVA of OD, decimal	0.71 (0.32)	0.91 (0.16)	<0.001^*^
BCVA of OS, decimal	0.76 (0.29)	0.92 (0.17)	<0.001^*^
IOP of OD, mmHg	14.01 (3.12)	14.30 (2.55)	0.365
IOP of OS, mmHg	13.87 (2.82)	14.16 (3.05)	0.367

*SBP, systolic blood pressure; DBP, diastolic blood pressure; HTN, hypertension; DM, diabetes mellitus; HLP, hyperlipidemia; CAD, coronary artery disease; AF, atrial fibrillation; FBG, fasting blood glucose; HbA1c, hemoglobin A1c; CHOL, cholesterol; TRIG, triglycerides; NEFA, non-esterified fatty acid; HDL, high-density lipoprotein; LDL, low-density lipoprotein; D-DI, d-dimmer; HCY, hemocyanin; BCVA, the best-corrected visual acuity; OD, oculus dextrus; OS, oculus sinister; IOP, intraocular pressure*.

a *Continuous variable as displayed as mean (SD) according to their distributions; Categoric data are displayed as a number (percentage)*.

b *HCY was unavailable for the following categories in the control group (n = 195)*.

* *p-value: stroke vs. control group statistically significant p < 0.05*.

### OCTA Parameters

#### Decreased Macular Capillary Plexus in Stroke

**Figure 1:**
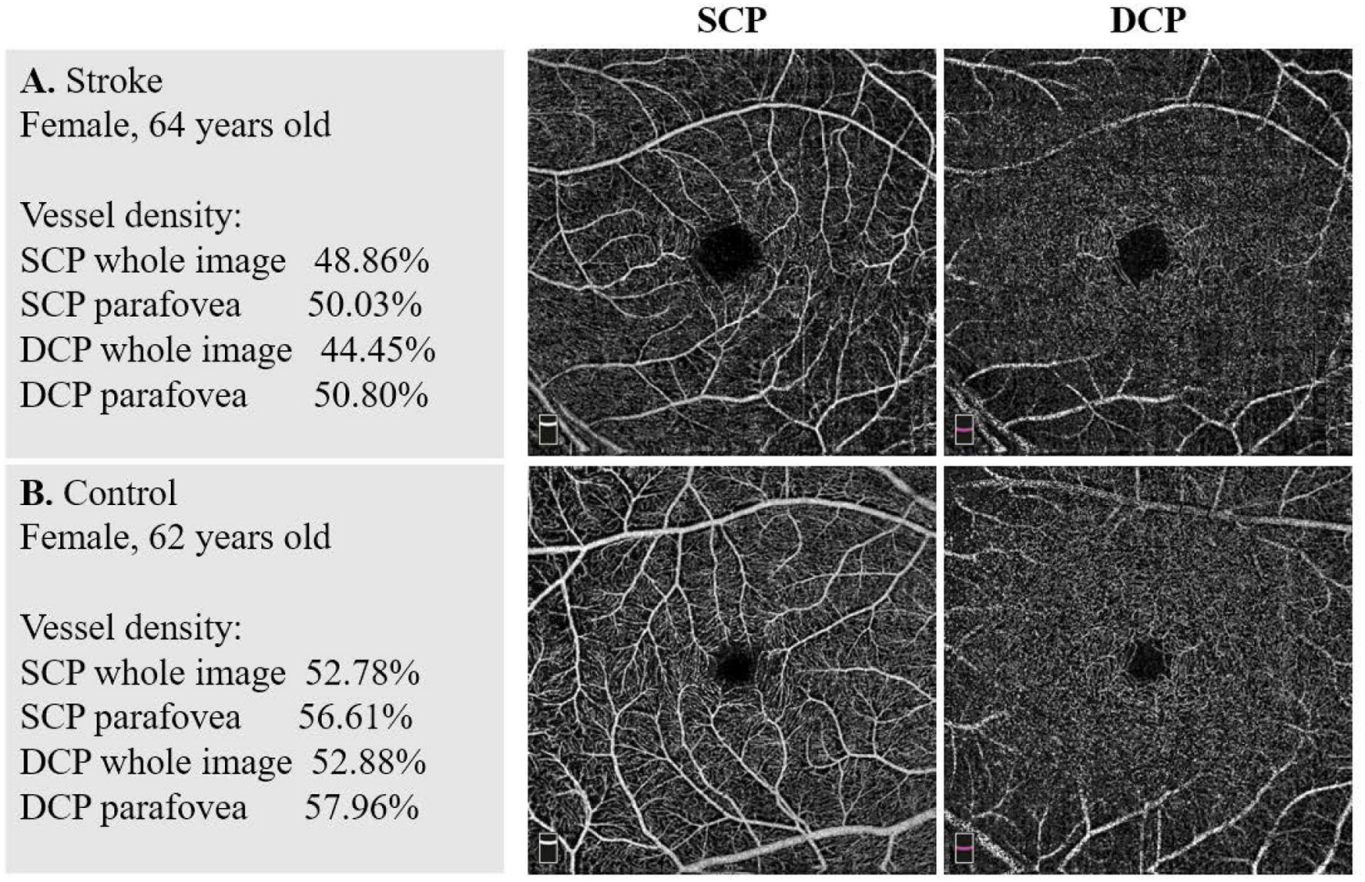
Example images of vascular density in the two groups. **(A)** A stroke patient's macular vessel density (VD) in superficial capillary plexus (SCP) and deep capillary plexus (DCP). **(B)** A non-stroke subject's macular VD in SCP and DCP. There were significant differences between the stroke group and the control group in VD of both SCP and DCP.

Comparisons of the macular VD of the two groups are shown in Table 2. In both SCP and DCP, the macular VD of every sector was significantly lower in the stroke group than in the control group after adjusting for the eight baseline characteristics (all *p* < 0.05). Typical example images of the macular VD in SCP and DCP of the two groups are shown in Figure 1. However, there were no significant differences of VD in both SCP and DCP between groups of stroke subtypes and locations, except for the Parafoveal VD in the DCP among different locations of stroke.

**Table 2:** Comparison of OCTA parameters in the two groups (*n* = 384).

**Macular VD**	**SCP**			**DCP**		
**Mean (SD), %**	**Stroke *n* = 189**	**Control *n* = 195**	***p*-value**	**Stroke *n* = 189**	**Control *n* = 195**	***p*-value**
Whole Image	47.45 (4.35)	49.44 (3.71)	<0.001^*^	47.64 (6.07)	50.75 (6.29)	<0.001^*^
S-Hemi	47.71 (4.37)	49.69 (3.82)	<0.001^*^	47.95 (6.13)	51.14 (6.53)	<0.001^*^
I-Hemi	47.16 (4.47)	49.19 (3.76)	0.001^*^	47.32 (6.29)	50.35 (6.22)	<0.001^*^
Fovea	17.73 (6.72)	18.55 (7.26)	0.001^*^	32.72 (7.36)	32.67 (7.45)	<0.001^*^
Parafovea	49.23 (5.56)	51.78 (4.67)	0.001^*^	52.26 (5.10)	55.17 (4.70)	0.003^*^
Para-S-Hemi	49.57 (5.85)	51.97 (4.98)	<0.001^*^	52.59 (5.22)	55.30 (5.08)	0.001^*^
Para-I-Hemi	48.89 (5.69)	51.44 (4.70)	0.004^*^	51.92 (5.45)	54.74 (4.58)	0.005^*^
Para-T	49.59 (5.96)	51.68 (4.70)	0.003^*^	53.27 (5.52)	55.84 (4.99)	<0.001^*^
Para-S	50.06 (6.77)	52.50 (5.28)	0.001^*^	51.82 (5.91)	54.83 (5.56)	0.009^*^
Para-N	48.12 (6.30)	50.76 (5.62)	0.001^*^	53.15 (5.74)	55.59 (4.60)	0.004^*^
Para-I	49.13 (6.20)	51.94 (4.88)	0.046^*^	50.79 (6.30)	53.90 (5.02)	0.025^*^
Perifovea	48.25 (4.56)	50.21 (3.71)	<0.001^*^	48.63 (6.73)	52.16 (6.76)	<0.001^*^
Peri-S-Hemi	48.47 (4.52)	50.40 (3.91)	<0.001^*^	48.98 (6.73)	52.55 (6.89)	<0.001^*^
Peri-I-Hemi	48.02 (4.82)	49.96 (3.81)	0.001^*^	48.26 (7.18)	51.53 (6.90)	<0.001^*^
Peri-T	44.75 (5.00)	46.67 (4.17)	0.003^*^	51.71 (6.55)	54.80 (6.21)	0.003^*^
Peri-S	48.74 (4.63)	50.57 (4.12)	<0.001^*^	48.24 (7.09)	51.67 (7.44)	<0.001^*^
Peri-N	51.74 (4.69)	53.62 (3.89)	0.003^*^	47.51 (7.32)	51.17 (7.45)	<0.001^*^
Peri-I	47.95 (5.11)	49.90 (4.08)	0.013^*^	47.15 (7.83)	50.50 (7.49)	0.001^*^
**PARAMETERS OF FAZ**						
FAZ area, mm^2^	0.32 (0.11)	0.34 (0.11)	<0.001^*^			
RERIM	2.21 (0.44)	2.26 (0.41)	<0.001^*^			
Acircularity index	1.12 (0.07)	1.11 (0.05)	0.008^*^			
FD-300	51.42 (5.92)	53.81 (5.32)	<0.001^*^			
**OPTIC DISC VD**						
Disc whole VD	54.61 (3.74)	55.50 (3.07)	0.006^*^			
Disc capillary VD	48.23 (3.54)	49.62 (2.89)	0.010^*^			

*VD, vessel density; SCP, superficial capillary plexus; DCP, deep capillary plexus; S-Hemi, superior-Hemi; I-Hemi, inferior-Hemi; T, temporal; S, superior; N, nasal; I, inferior; FAZ, foveal avascular zone; RERIM, the FAZ perimeter; FD-300, the VD within a 300-μm wide ring surrounding the FAZ*.

*Stroke vs. control group: adjusted for sex, SBP, DBP, history of smoking, HbA1c, CHOL, HDL, BCVA of enrolled eyes*.

* *p-value: stroke vs. control group statistically significant p < 0.05*.

#### Irregular FAZ in Stroke

Comparisons of the FAZ metrics of the two groups are shown in Table 2. The FAZ area, FAZ perimeter, and FD-300 were significantly decreased in the stroke group compared to the control group after adjusting for the eight baseline characteristics (all *p* < 0.001). The FAZ acircularity index, representing the irregularity of FAZ, was significantly greater in the stroke group after the same adjustment (stroke: 1.12 ± 0.07, controls: 1.11 ± 0.05, *p* = 0.008). There were no significant differences of FD-300 between groups of stroke subtypes and locations.

#### Decreased Peripapillary Capillary Plexus in Stroke

For the peripapillary area, a significant decrease in the whole image VD (stroke: 54.61 ± 3.74%, controls: 55.50 ± 3.07%, *p* = 0.06) and capillary VD (stroke: 48.23 ± 3.54%, controls: 49.62 ± 2.89%, *p* = 0.010) were found in the stroke group compared to the control group after adjusting for the eight baseline characteristics (Table 2). There were no significant differences of the peripapillary VD between groups of stroke subtypes and locations.

### Logistic Regression Analysis

Univariate regression analyses (Table 3) showed that participants of the female gender (OR: 0.179), with a higher CHOL (OR: 0.688), higher HDL (OR: 0.023), and better BCVA (OR: 0.037) had lower odds of being classified as stroke, while those with a higher SBP (OR: 1.034), higher DBP (OR: 1.050), and a history of smoking (OR: 2.137) were associated with higher odds of being classified as stroke. As for the OCTA parameters, participants with a higher VD of SCP and DCP in most of the sectors (OR < 1.000; all *p* < 0.001 except for fovea), a higher FD-300 of the FAZ (OR: 0.431), and a higher VD of the optic disc (capillary, OR: 0.518) were found to have lower odds of being classified as stroke.

**Table 3:** Risk factors for stroke using binary logistic regression analysis (*n* = 384).

**Characteristics**	**Univariate model, OR (95% CI)**	***p*-value**	**Multivariate model, OR (95% CI)**	***p*-value**
**BASELINE CHARACTERISTICS**				
Sex	0.179 (0.115–0.278)	<0.001^*^	0.191 (0.073–0.499)	0.001^*^
SBP at admission, mmHg	1.034 (1.020–1.048)	<0.001^*^	N/A	N/A
DBP at admission, mmHg	1.050 (1.026–1.075)	<0.001^*^	1.051 (1.005–1.098)	0.029
History of smoking	2.137 (1.289–3.543)	0.003^*^	N/A	N/A
HbA1c, %	N/A	0.068	N/A	N/A
CHOL, mmol/L	0.688 (0.562–0.844)	<0.001^*^	N/A	N/A
HDL, mmol/L	0.023 (0.007–0.071)	<0.001^*^	0.028 (0.003–0.277)	0.002^*^
BCVA of enrolled eyes	0.037 (0.013–0.112)	<0.001^*^	0.012 (0.001–0.130)	<0.001^*^
**MACULAR VD OF SCP**				
Whole Image, %	0.420 (0.279–0.632)	<0.001^*^	N/A	N/A
S-Hemi, %	0.376 (0.249–0.568)	<0.001^*^	N/A	N/A
I-Hemi, %	0.420 (0.279–0.632)	<0.001^*^	N/A	N/A
Fovea, %	N/A	0.994	N/A	N/A
Parafovea, %	0.368 (0.244–0.556)	<0.001^*^	N/A	N/A
Para-S-Hemi, %	0.429 (0.285–0.646)	<0.001^*^	N/A	N/A
Para-I-Hemi, %	0.458 (0.305–0.689)	<0.001^*^	N/A	N/A
Para-T, %	0.543 (0.363–0.815)	0.003^*^	N/A	N/A
Para-S, %	0.458 (0.305–0.689)	<0.001^*^	N/A	N/A
Para-N, %	0.458 (0.305–0.689)	<0.001^*^	N/A	N/A
Para-I, %	0.499 (0.333–0.749)	0.001^*^	N/A	N/A
Perifovea, %	0.448 (0.298–0.674)	<0.001^*^	N/A	N/A
Peri-S-Hemi, %	0.352 (0.233–0.532)	<0.001^*^	N/A	N/A
Peri-I-Hemi, %	0.452 (0.300–0.680)	<0.001^*^	N/A	N/A
Peri-T, %	0.396 (0.263–0.598)	<0.001^*^	N/A	N/A
Peri-S, %	0.439 (0.291–0.660)	<0.001^*^	N/A	N/A
Peri-N, %	0.456 (0.303–0.686)	<0.001^*^	N/A	N/A
Peri-I, %	0.491 (0.326–0.738)	0.001^*^	N/A	N/A
**MACULAR VD OF DCP**				
Whole image, %	0.402 (0.267–0.606)	<0.001^*^	N/A	N/A
S-Hemi, %	0.368 (0.244–0.556)	<0.001^*^	N/A	N/A
I-Hemi, %	0.420 (0.279–0.632)	<0.001^*^	N/A	N/A
Fovea, %	N/A	0.257	N/A	N/A
Parafovea, %	0.308 (0.203–0.467)	<0.001^*^	N/A	N/A
Para-S-Hemi, %	0.300 (0.198–0.457)	<0.001^*^	0.169 (0.066–0.432)	<0.001^*^
Para-I-Hemi, %	0.368 (0.244–0.556)	<0.001^*^	N/A	N/A
Para-T, %	0.385 (0.255–0.580)	<0.001^*^	N/A	N/A
Para-S, %	0.322 (0.212–0.488)	<0.001^*^	N/A	N/A
Para-N, %	0.385 (0.255–0.580)	<0.001^*^	N/A	N/A
Para-I, %	0.321 (0.212–0.488)	<0.001^*^	N/A	N/A
Perifovea, %	0.385 (0.255–0.580)	<0.001^*^	N/A	N/A
Peri-S-Hemi, %	0.344 (0.227–0.521)	<0.001^*^	N/A	N/A
Peri-I-Hemi, %	0.433 (0.287–0.652)	<0.001^*^	N/A	N/A
Peri-T, %	0.414 (0.275–0.624)	<0.001^*^	N/A	N/A
Peri-S, %	0.458 (0.305–0.689)	<0.001^*^	N/A	N/A
Peri-N, %	0.383 (0.253–0.578)	<0.001^*^	N/A	N/A
Peri-I, %	0.450 (0.299–0.678)	<0.001^*^	N/A	N/A
**PARAMETERS OF FAZ**				N/A
FAZ area, mm^2^	0.663 (0.443–0.993)	0.046^*^	N/A	N/A
RERIM	N/A	0.073	N/A	N/A
Acircularity index	N/A	0.447	N/A	N/A
FD-300, %	0.431 (0.286–0.649)	<0.001^*^	N/A	N/A
**OPTIC DISC VD**				
Disc whole VD, %	N/A	0.163	N/A	N/A
Disc capillary VD, %	0.518 (0.344–0.781)	0.002^*^	N/A	N/A

*OR, odds ratios; CI, confidence interval; SBP, systolic blood pressure; N/A, not applicable; DBP, diastolic blood pressure; HbA1c, hemoglobin A1c; CHOL, cholesterol; HDL, high-density lipoprotein; BCVA, the best-corrected visual acuity; VD, vessel density; SCP, superficial capillary plexus; DCP, deep capillary plexus; S-Hemi, superior-Hemi; I-Hemi, inferior-Hemi; T, temporal; S, superior; N, nasal; I, inferior; FAZ, foveal avascular zone; RERIM, the FAZ perimeter; FD-300, the VD within a 300-μm wide ring surrounding the FAZ*.

* *p < 0.05 from binary logistic regression analysis*.

Multivariate regression analyses (Table 3) showed that female gender (OR: 0.191), HDL > 1.04 mmol/L (median; OR: 0.028), BCVA > 0.9 (median; OR: 0.012), and the parafovea-superior-hemi VD of DCP > 54.53% (median; OR: 0.169) were independently associated with lower odds of the participants being classified as stroke, while DBP > 82.0 mmHg (median; OR: 1.051) was independently associated with higher odds of the participants being classified as stroke.

### ROC Analysis

**Figure 2:**
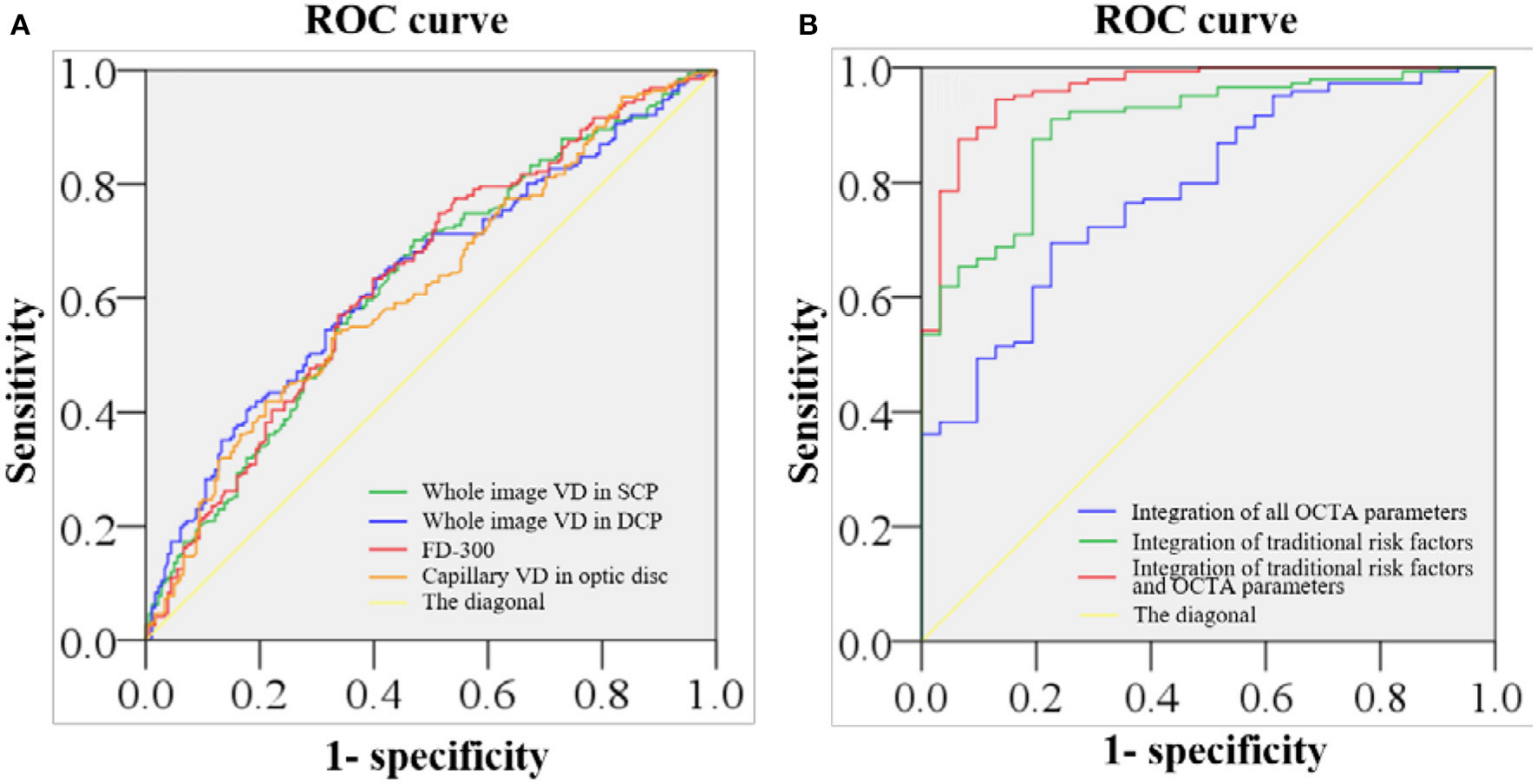
ROC of retinal VD in distinguishing stroke from non-stroke. **(A)** The area under the receiver operating characteristic curve (AUC) of the macular VD of SCP, the macular VD of DCP, the VD within a 300-μm wide ring surrounding the foveal avascular zone (FAZ) (FD-300), and the optic disc VD of capillary is 0.630, 0.639, 0.638, and 0.619, respectively. **(B)** The AUC of the integration of all optical coherence tomography angiography (OCTA) parameters is 0.787, with the sensitivity of 0.694 and the specificity of 0.774. The AUC of the integration of traditional risk factors is 0.894, with the sensitivity of 0.89 and the specificity of 0.774. The AUC of the integration of all OCTA parameters and traditional risk factors 0.962, with the sensitivity of 0.944 and the specificity of 0.871.

The ROC showed the ability to distinguish patients with stroke from the control subjects (Figure 2). The AUC of the macular VD in SCP, the macular VD in DCP, FD-300, and the capillary VD of optic disc was 0.630, 0.639, 0.638, and 0.619, respectively. The AUC of the integration of all OCTA parameters was 0.787, with the sensitivity of 0.694 and the specificity of 0.774. The AUC of the integration of traditional risk factors was 0.894, with the sensitivity of 0.89 and the specificity of 0.774. The AUC of the integration of all OCTA parameters and traditional risk factors was 0.962, with the sensitivity of 0.944 and the specificity of 0.871.

## Discussion

Despite being extensively studied, the change of microvasculature in the pathogenesis and evolution of stroke remains unclear (Cheung et al., 2017; O'Donnell and Yuan, 2018). Retinal vessels offer a unique “window” to non-invasively investigate the change of the microcirculation in cerebrovascular diseases because retinal vasculature shares many common anatomical and physiological characteristics with the cerebral vasculature (London et al., 2013; Jung et al., 2019; McGrory et al., 2019). In this study, we used OCTA to quantitatively measure the retinal microvasculature in patients with stroke. We found that the retinal VD in macula and optic disc was decreased in patients with stroke independently of the traditional risk factors of stroke.

In the present study, the retinal capillary rarefaction in both macular and optic disc vascular plexus was observed in patients with stroke, suggesting possible retinal vascular pathologies in stroke. Previous studies had reported associations between strokes and retinal microvascular lesions, such as a narrower arteriolar caliber, arteriovenous nicking, and a wider venular caliber (Kwa et al., 2002; Ikram et al., 2004, 2006; De Silva et al., 2009; Kaushik et al., 2009; McGeechan et al., 2009; Baker et al., 2010; Vuong et al., 2015). These studies showed that retinal vascular abnormalities found in patients with stroke might be associated with systemic risk factors such as hypertension. Previous studies also observed hypertension-evoked remodeling of cerebral vessels leading to hypoperfusion and exacerbation of ischemia of the brain (Morrison and Filosa, 2019). In retina, similar pathological changes may also exist in patients with hypertension. Retinal vessel morphology has been noticed to be altered in hypertension as the retinal VD was demonstrated to be decreased in patients with hypertension (Peng et al., 2020).

There are other possible mechanisms of retinal capillary rarefaction since the retinal hypoperfusion in patients with stroke remains significant after the adjustment of the traditional risk factors of stroke in our study. Firstly, the neurovascular unit (NVU) theory has been proposed as a mechanism of stroke in recent years. The NVU consists of several types of cells and extracellular matrices, and acts as an integrated system for the regulation of cerebral blood flow (Morrison and Filosa, 2019). According to the NVU theory, ischemic stroke is associated with the damage to the integrate function of the NVU (Steliga et al., 2020). The NVU is also proposed to exist in retina, and damage to the retinal NVU has been observed in cardiovascular diseases (Simó et al., 2018; Peng et al., 2020). Secondly, stroke potentiates a cascade of ischemic events that lead to the impairment of cerebral NVU resulting in blood-brain barrier (BBB) damage. Astrocytes, microglia, and pericytes provide a structural and functional support to the BBB. Ischemic stroke leads to cerebral NVU remodeling due to the detachment of astrocytic endfoot, pericyte detachment, vasconstriction, the dysfunction of neurovascular coupling, the activation of microglia, and the rupture of a blood vessel (Jayaraj et al., 2019). Composition of the retinal NVU is similar to that of the cerebral NVU (André et al., 2012). Thus, the retinal NVU can also be damaged by the factors impairing the cerebral NVU in stroke. Finally, the activation of neuroinflammation could be another possible mechanism of retinal capillary rarefaction. For instance, active neutrophils lead to microvessel obstruction, reactive oxygen species (ROS) production, and release of matrix metalloproteinases (MMPs), and other proteolytic enzymes that cause damage to the endothelial cell membrane and increase BBB permeability, contributing to BBB damage and neuroinflammation (Geng et al., 2019). In addition, T lymphocytes facilitate the adhesion of platelets and leukocytes to the vascular endothelium causing thrombo-inflammation by activating proinflammatory pathways (Feng et al., 2017). The abovementioned activation of neuroinflammation pathways is likely to be responsible for inducing abnormalities in retinal and cerebral vasculatures. These similar pathophysiological processes might be the mechanisms that underlie the decreased retinal VD of patients with stroke, given that retinal and cerebral microvasculatures share many morphological, physiological, and pathogenic properties.

In the present study, we also found that BCVA was decreased in patients with stroke, which might be related to cerebral damages and retinal hypoperfusion. On one hand, a high incidence and prevalence of visual problems in patients with stroke have been reported (Rowe, 2017). The visual problems include reduced central visual acuity, visual field loss, ocular motility disorders, visual perceptual disorders, etc., which cause ischemic and hypoxic damages to the brain tissue (Faieta and Page, 2016; Rowe, 2017). On the other hand, the retinal hypoperfusion, especially in the DCP layer, can also lead to a decreased BCVA. The deep vascular plexus of retina is mainly enveloped by the pericytes that are sensitive to hypoxia secondary to hypoperfusion (Flower, 1988; Hardy et al., 2000). Under hypoxic conditions, the DCP is prone to pathological changes, such as the loss of pericytes (Trost et al., 2019). In addition, the capillaries of the DCP are vital for nutrition and oxygen support to the inner nuclear layer (INL) and the outer plexiform layer (OPL), where the photoreceptor axon terminals form ribbon synapses with bipolar cells and horizontal cells (Birol et al., 2007). The INL and OPL are located near a watershed zone between retinal and the choroidal vasculatures, where the nutrition and oxygen supply is mainly provided by the DCP (Heidelberger et al., 2005). Therefore, hypoperfusion in the DCP can cause nutrition and oxygen deficiency in the synaptic connections. This might be one of the reasons why the BCVA decreased in patients with stroke.

Moreover, we also found a smaller area and perimeter of the FAZ in patients with stroke. Our findings seemed to be different from the other studies showing an enlarged FAZ area in patients with diabetes mellitus, which is one of the major risk factors of stroke (Choi et al., 2020). One of the possible reasons was the sex distribution of our patients with stroke. A previous study showed that the FAZ area was smaller in males compared to females (Gómez-Ulla et al., 2019). Since stroke is more commonly seen in male patients, the proportion of males was significantly higher in the stroke group (75.13%) compared to the control group (34.87%). Therefore, the smaller FAZ in the stroke group might be due to there being more male patients in the group. Nevertheless, further studies are needed to reveal the exact mechanisms of the smaller FAZ in patients with stroke. In the present study, we also found increased irregularity of the FAZ in patients with stroke, indicated by an increased FAZ acircularity index in the stroke group. The pathological mechanisms underlying the increased irregularity of the FAZ are multifactorial, with capillary occlusion, hemodynamic disturbances, and endothelial dysfunction playing their roles in the formation of these capillary abnormalities (Inanc et al., 2019). It has been shown that the FAZ acircularity index is increased in patients with more severe DR (de Carlo et al., 2015; Krawitz et al., 2017), which is suggested to be used to monitor the progression of DR. According to the results of our study, the FAZ acircularity index could also be used for the monitoring of stroke.

Retina is a highly metabolically active tissue and one of the tissues with the highest oxygen consumption in our body (Benjamin et al., 2017). Therefore, retina is highly sensitive to hypoperfusion and hypoxia. It is found that the retinal VD is decreased in hypertension (Lee et al., 2019), coronary heart disease (Wang et al., 2019), congenital heart disease (Li et al., 2020), obstructive sleep apnea syndrome (Moyal et al., 2018), etc. Similarly, in the current study, we also observed a significantly reduced retinal VD in patients with stroke. Multivariate logistic regression analyses found that participants with a decreased parafovea VD of DCP were less likely to be classified as stroke. Furthermore, the ROC analysis showed that the integration of all OCTA parameters and the traditional risk factors of stroke could provide good identification of stroke evidenced by an AUC of 0.962. Since OCTA measurements in the present study were performed after a stroke attack, it was possible that the retinal VD was decreased over time after stroke. In further analyses, we showed that the OCTA parameters in the stroke group were not correlated to the time interval between stroke attacks (the first attack and the last attack) and the OCTA examination. The OCTA parameters were also not significantly different between patients with stroke with a shorter time interval and those with a longer time interval (Supplementary Tables 1, 2). Although at this point, we could not rule out the possibility of a decrease in poststroke retinal VD, a reduced retinal VD in patients with stroke was unlikely all due to poststroke changes, and pre-stroke changes could also play a role. Overall, our results showed that the OCTA parameters could provide additional information about microvascular changes in stroke beyond traditional biomarkers, but our study was just the prelude to a future longitudinal and prospective study, which could establish with a greater certainty of the usefulness of OCTA parameters as potential predictive markers of stroke.

To our best knowledge, the present study is the first one to evaluate the retinal microvasculature in patients with stroke using OCTA. On one hand, our study suggests that retinal microvascular abnormalities in stroke can be reliably detected and accurately quantified by using OCTA, which is non-invasive and cost-effective without the radiation exposure from CT or the invasive operation from DSA. On the other hand, an automatic segmentation of the retina using OCTA allows a direct observation and quantification of retinal vessels in different retinal layers and regions. In fact, the high resolution three-dimensional scanning of OCTA can provide us more accurate information about retinal microvascular abnormalities than the two-dimensional retinal photography (Or et al., 2018). Moreover, a direct quantification of retinal vessels using the built-in software of OCTA reduces quadric measurement errors. Taken together, similar pathological changes between retinal and cerebral vasculatures in stroke and an accurate quantification of retinal vessels using OCTA have made it possible for us to provide a more comprehensive evaluation of the microvasculature in stroke. However, future longitudinal and prospective studies are needed to further verify the accuracy of the retinal VD in predicting the stroke events.

The main strength of our prospective study is the use of a state-of-the-art technology, OCTA, to observe retinal microvascular changes in patients with stroke. Nonetheless, we acknowledge several limitations in our research. Firstly, for the sake of safety, patients with unstable vital signs were excluded, which could have inevitably led to a selection bias. In future, portable OCTA instruments might be able to deal with such problems. Secondly, fewer female patients than male patients with stroke were included. This may be due to the fact that stroke is more common among males (Reeves et al., 2008). Males are also more easily subjected to the risk factors of stroke such as smoking, drinking, and other unhealthy behaviors (Li et al., 2016). Therefore, a sex selection bias was inevitable in our study. Thirdly, the National Institute of Health stroke scale (NIHSS) should be included to investigate the relationship between the severity of stroke and retinal blood-flow changes in future studies. Finally, the ultrahigh resolution 6 × 6 mm OCTA could only capture retinal vessels at the posterior pole, which would limit our understanding of the microvascular changes in the peripheral retina in patients with stroke. Future improvements in wild field OCTA imaging can be expected to address this limitation.

## Conclusions

Our study indicates that retinal VD is decreased in patients with stroke independently of the traditional risk factors of stroke. These findings suggest that the OCTA parameters can provide additional information about microvascular changes of stroke, but future longitudinal and prospective studies are needed for greater certainty of its usefulness as a potential predictive marker.

## Data Availability Statement

The original contributions presented in the study are included in the article/Supplementary Material; further inquiries can be directed to the corresponding author/s.

## Ethics Statement

The studies involving human participants were reviewed and approved by the institutional review board of the Guangdong Provincial People's Hospital, Guangzhou, China [registration number: GDREC2018148H(R1)]. The patients/participants provided their written informed consent to participate in this study. Written informed consent was obtained from the individual(s) for the publication of any potentially identifiable images or data included in this article.

## Author Contributions

BL, LW, HY, and XY: conception and design. BL, GM, YX, BZ, YL, PZ, XZ, ZL, HK, GW, ZD, YF, MH, and HY: data collection, analysis, and interpretation. BL, YH, GM, and HY: drafting the article. BL, YH, GM, LW, HY, and XY: revising it critically for important intellectual content. All authors read and approved the final manuscript.

## Conflict of Interest

The authors declare that the research was conducted in the absence of any commercial or financial relationships that could be construed as a potential conflict of interest.
