# Supplementary material for: Reduced Retinal Microvascular Perfusion in Patients With Stroke Detected by Optical Coherence Tomography Angiography
Source: Front Aging Neurosci. 2021 Apr 13;13:628336. doi: 10.3389/fnagi.2021.628336 (PMC8078175; doi:10.3389/fnagi.2021.628336)
Supplement: Supplementary file 1 [file Data_Sheet_1.PDF]

Supplement table 1. Correlations between OCTA parameters and duration of stroke

Macular VD, % (n = 189)	Duration of stroke, day			
	From the first attack		From the last attack	
	r value	*p* value	*r* value	*p* value
**SCP**				
Whole Image	0.053	0.469	0.106	0.145
S-Hemi	0.051	0.484	0.102	0.162
I-Hemi	0.037	0.610	0.089	0.224
Fovea	0.036	0.623	-0.062	0.394
Parafovea	0.006	0.932	0.033	0.654
Para-S-Hemi	-0.009	0.899	0.014	0.852
Para-I-Hemi	0.012	0.866	0.040	0.589
Para-T	0.010	0.889	0.048	0.515
Para-S	0.069	0.348	0.020	0.781
Para-N	0.005	0.944	-0.007	0.928
Para-I	0.009	0.907	0.051	0.489
Perifovea	0.049	0.506	0.094	0.199
Peri-S-Hemi	0.063	0.390	0.105	0.152
Peri-I-Hemi	0.038	0.607	0.078	0.285
Peri-T	0.039	0.597	0.053	0.474
Peri-S	0.050	0.494	0.105	0.152
Peri-N	0.041	0.573	0.071	0.331
Peri-I	0.061	0.407	0.100	0.174
**DCP**				
Whole Image	-0.017	0.820	0.047	0.521
S-Hemi	-0.024	0.745	0.051	0.489
I-Hemi	-0.026	0.724	-0.106	0.147
Fovea	-0.032	0.661	-0.090	0.221
Parafovea	-0.027	0.717	0.009	0.904
Para-S-Hemi	-0.033	0.650	-0.003	0.965
Para-I-Hemi	-0.028	0.705	0.009	0.907
Para-T	0.015	0.834	0.061	0.407
Para-S	-0.054	0.461	-0.028	0.702
Para-N	-0.033	0.650	-0.028	0.704
Para-I	-0.032	0.658	0.017	0.821
Perifovea	-0.018	0.807	0.030	0.683
Peri-S-Hemi	-0.020	0.788	0.032	0.664
Peri-I-Hemi	-0.014	0.845	0.033	0.651
Peri-T	0.021	0.773	0.052	0.480
Peri-S	-0.059	0.418	0.027	0.710
Peri-N	-0.052	0.481	-0.022	0.769
Peri-I	0.014	0.850	0.065	0.380
**FAZ**				
FAZ area, mm^2	0.026	0.729	0.014	0.850

RERIM	0.029	0.695	0.040	0.588
Acircularity index	0.038	0.603	0.058	0.433
FD-300	0.013	0.860	0.004	0.959
**Optic disc**				
Disc whole	0.060	0.421	0.050	0.499
Disc capillary	0.085	0.251	0.070	0.346

Abbreviations: OCTA, optical coherence tomography angiography; VD, vessel density; SCP, superficial capillary plexus; DCP, deep capillary plexus; S-Hemi, superior-Hemi; I-Hemi, inferior-Hemi; T, temporal; S, superior; N, nasal; I: inferior; FAZ, foveal avascular zone; RERIM, the FAZ perimeter; FD-300, the vessel density within a 300-μm wide ring surrounding the FAZ.

OCTA parameters in the stroke group were not correlated to the time interval between stroke attacks (the first attack and the last attack) and OCTA examination.

* $p < 0.05$ was statistically significant by Spearman's correlation analysis.

Supplement table 2. Comparison of OCTA parameters in the stroke patients

**Macular VD, Mean (SD), % (n = 189)**	**Duration of stroke, day**					
	From the first attack			From the last attack		
	≤ median	> median	*p1* value	≤ median	> median	*p2* value
**SCP**						
Whole Image	47.03 ± 4.08	47.88 ± 4.40	0.170	47.06 ± 4.29	47.85 ± 4.20	0.205
S-Hemi	47.29 ± 3.99	48.14 ± 4.53	0.169	47.32 ± 4.25	48.10 ± 4.30	0.211
I-Hemi	46.74 ± 4.36	47.59 ± 4.38	0.186	46.76 ± 4.48	47.57 ± 4.25	0.208
Fovea	18.03 ± 6.76	17.90 ± 6.78	0.896	18.42 ± 6.91	17.50 ± 6.59	0.346
Parafovea	48.92 ± 5.41	49.54 ± 5.49	0.435	49.06 ± 5.43	49.41 ± 5.47	0.660
Para-S-Hemi	49.15 ± 5.92	49.99 ± 5.52	0.318	49.39 ± 5.79	49.75 ± 5.67	0.661
Para-I-Hemi	48.69 ± 5.37	49.10 ± 5.78	0.616	48.72 ± 5.58	49.06 ± 5.58	0.681
Para-T	49.22 ± 5.95	49.95 ± 5.71	0.393	49.24 ± 5.90	49.94 ± 5.77	0.412
Para-S	50.00 ± 6.40	50.82 ± 6.36	0.379	50.57 ± 6.19	50.24 ± 6.60	0.720
Para-N	48.01 ± 5.62	48.23 ± 6.70	0.810	48.19 ± 6.03	48.05 ± 6.33	0.876
Para-I	48.74 ± 6.50	49.56 ± 5.63	0.356	48.91 ± 6.24	49.38 ± 5.94	0.599
Perifovea	47.83 ± 4.31	48.68 ± 4.60	0.190	47.90 ± 4.48	48.61 ± 4.44	0.273
Peri-S-Hemi	48.04 ± 4.08	48.89 ± 4.73	0.188	48.08 ± 4.35	48.85 ± 4.49	0.233
Peri-I-Hemi	47.57 ± 4.81	48.46 ± 4.62	0.198	47.67 ± 4.87	48.36 ± 4.57	0.317
Peri-T	44.27 ± 4.85	45.24 ± 4.91	0.173	44.57 ± 4.76	44.93 ± 5.04	0.616
Peri-S	48.40 ± 3.90	49.08 ± 5.09	0.305	48.33 ± 4.45	49.14 ± 4.60	0.219
Peri-N	51.23 ± 4.81	52.27 ± 4.31	0.121	51.32 ± 4.78	52.16 ± 4.38	0.211
Peri-I	47.44 ± 5.12	48.47 ± 4.84	0.160	47.47 ± 5.21	48.42 ± 4.75	0.195
**DCP**						
Whole Image	47.58 ± 5.48	47.70 ± 6.40	0.885	47.19 ± 5.42	48.09 ± 6.42	0.296
S-Hemi	47.96 ± 5.31	47.94 ± 6.65	0.988	47.49 ± 5.30	48.41 ± 6.63	0.293
I-Hemi	47.65 ± 6.41	47.97 ± 6.21	0.725	48.34 ± 6.12	47.28 ± 6.46	0.247
Fovea	32.91 ± 7.66	32.37 ± 6.52	0.605	33.09 ± 7.73	32.19 ± 6.41	0.383

Parafovea	52.29 ± 4.12	52.22 ± 5.76	0.920	52.00 ± 4.42	52.52 ± 5.52	0.478
Para-S-Hemi	52.49 ± 4.49	52.69 ± 5.68	0.795	52.28 ± 4.69	52.91 ± 5.50	0.395
Para-I-Hemi	52.09 ± 4.38	51.75 ± 6.16	0.663	51.72 ± 4.77	52.12 ± 5.86	0.606
Para-T	53.00 ± 5.16	53.54 ± 5.64	0.498	52.76 ± 5.38	53.78 ± 5.40	0.196
Para-S	51.97 ± 4.93	51.67 ± 6.56	0.715	51.76 ± 5.18	51.89 ± 6.37	0.880
Para-N	53.19 ± 5.06	53.11 ± 6.15	0.924	52.81 ± 5.64	53.49 ± 5.59	0.401
Para-I	51.00 ± 5.25	50.56 ± 6.98	0.625	50.67 ± 5.31	50.90 ± 6.94	0.797
Perifovea	48.57 ± 6.14	48.68 ± 7.03	0.906	48.22 ± 5.98	49.04 ± 7.15	0.389
Peri-S-Hemi	49.01 ± 5.82	48.95 ± 7.31	0.945	48.61 ± 5.72	49.35 ± 7.37	0.439
Peri-I-Hemi	48.13 ± 6.94	48.40 ± 7.14	0.790	47.80 ± 6.75	48.73 ± 7.29	0.364
Peri-T	51.21 ± 6.59	52.22 ± 6.21	0.279	51.30 ± 6.09	52.13 ± 6.72	0.372
Peri-S	48.54 ± 5.84	47.94 ± 7.91	0.551	47.91 ± 5.87	48.57 ± 7.89	0.511
Peri-N	47.66 ± 6.74	47.37 ± 7.60	0.779	47.05 ± 6.77	47.97 ± 7.54	0.379
Peri-I	46.89 ± 7.56	47.41 ± 7.79	0.648	46.56 ± 7.55	47.73 ± 7.76	0.297
**FAZ**						
FAZ area, mm^2	0.32 ± 0.12	0.32 ± 0.10	0.834	0.32 ± 0.13	0.32 ± 0.09	0.858
RERIM	2.21 ± 0.46	2.21 ± 0.39	0.980	2.20 ± 0.50	2.23 ± 0.34	0.656
Acircularity index	1.12 ± 0.06	1.12 ± 0.07	0.733	1.12 ± 0.06	1.13 ± 0.07	0.413
FD-300	51.33 ± 5.18	51.50 ± 6.37	0.845	51.02 ± 6.52	51.82 ± 4.94	0.348
**Optic disc**						
Disc whole	54.23 ± 4.29	54.95 ± 2.89	0.187	54.22 ± 4.15	54.96 ± 3.08	0.175
Disc capillary	47.75 ± 4.08	48.64 ± 2.67	0.081	47.79 ± 3.96	48.60 ± 2.87	0.111

Abbreviations: OCTA, optical coherence tomography angiography; VD, vessel density; SCP, superficial capillary plexus; DCP, deep capillary plexus; S-Hemi, superior-Hemi; I-Hemi, inferior-Hemi; T, temporal; S, superior; N, nasal; I: inferior; FAZ, foveal avascular zone; RERIM, the FAZ perimeter; FD-300, the vessel density within a 300-μm wide ring surrounding the FAZ.

The stroke patients were divided into two groups based on the median of the duration. The OCTA parameters were not significantly different between the stroke patients with shorter time interval and those with longer time interval.

* $p < 0.05$ was statistically significant by independent, two-tailed student's *t* tests.
